# Woman’s Apparel

**Published:** 1875-04

**Authors:** 


### WOMAN'S APPAREL.

There are spasmodic efforts at reform in woman's dress, but we are unable to learn that they result in anything tangible. We receive enthusiastic letters from women who state that they find ease and freedom in an abandonment of corsets; in the use of shoulder-suspenders for skirts; in giving up garters which encircle the limb, and substituting straps connecting with a waist-band; in the adoption of some new form of under-garment; but no woman has yet told us that she uniformly wears short skirts in the street, and defies the observations of the multitude.

This is the one reform most needed. From this must come the redemption of the sex from much of its feebleness and misery. Through a shortening of the skirt must proceed a lengthening of life, a strengthening of the vital forces, an enlargement of physical and mental capacity, and an accession of power, which, we venture to assert, will never be attained by any other means.

It is not in the drawing-room or carriage, that long skirts do harm, nor do we seek to banish them from these localities. To display them at home or on dress occasions, is scarcely more than a matter of convenience. That "trains" and "demi-trains" and trailing skirts, are often ornamental and graceful, when worn by a conspicuous figure, over clean rich carpets, can not be denied; but it will be admitted that under the most favorable circumstances they are awkward to handle, cumbersome to the wearer, greatly in the way of others, and generally wasteful and extravagant. Very long skirts, even when most appropriately worn, induce selfishness, because the wearer must soon reach the point at which concern for the comfort of those by whom she is surrounded, is a thing of secondary importance. The display is the chief thing; the annoyance of those whose space in the ball-room or at the reception, is curtailed, receives no consideration. The unlucky creature who is compelled to tread upon the yards of dragging silk, is secretly if not openly anathematized; and it is safe to say that no woman ever wore a long train at a crowded party who did not go home irritated in temper, flushed with indignation, and strengthened in selfishness and vanity. The pure, and sweet, and simple, and childlike, do not affect trains, because they find that they quickly lose those characteristics if they wear them. Dragging skirts do not become them any more than they become the little children whose manners and characteristics remain with the simple, childlike class of women.

But these are minor matters. We confess to a sense of admiration as we look upon the gorgeous silken train, when gracefully borne, by one who is physically and pecuniarily able to bear it. We do not seek to ostracise it. We know how futile it would be to attempt to banish it from fashionable circles, and we gracefully submit to it in all proper places.

But we have to deal with the skirts that drag and drabble through the mud and dust of the public thoroughfare; which destroy the grace and ease of locomotion; which offend the senses of all who follow after; which, if at all protected from the mire of the streets, pinion the arms in such a manner as to make free respiration impossible; which induce palpitation of the heart, shortness of breath, and a whole catalogue of diseases; which, in fine, entail much of the existing weakness and ill health upon the female sex, and tend surely to a lessening of the span of human life. This is the great error which we combat—the evil which we are struggling earnestly to reform.

Motion is life; inertia is death. We must exercise to be well. The limb which is not used perishes; the muscle upon which no demands are made, losses its power to perform. The woman whose arms and hands are employed in holding her skirts out of the dirt of the street, can not secure for them the benefits of that salutary exercise which sets the blood all aglow and vitalizes it; which strengthens the muscles of the chest; increases by the most salutary means the involuntary action of the heart and lungs; imparts a genuine vigor to all the internal organs; gives to the system the power to ward off disease; increases assimilation and nutrition; and, by furthering all the natural processes of the organism, secures its healthful, normal action, and all the marvellous possibilities of both body and mind, which attend upon the perfect physical condition.

The mind must ever sympathise with the condition of the body. If the one works in fetters the other will lack freedom and expansion. If we would make our women great and good and strong and noble, we must begin at the beginning. The beginning is at the foundation of animal life. This must be vigorous and strong. Without a solid basis of wholesome muscle, there is absolutely nothing to be relied upon. It need not be massive, but it must be fine-grained and closely compacted. The simple truth is, that our women can not have it, can not retain it, if hampered by ill-devised costume. Exercise implies freedom; and there can be no freedom worth mentioning in which the hands and arms do not bear a part.

The most sensible woman whom it is our privilege to know, has, within the past week, become convinced of the folly and wickedness of wearing, out of doors, skirts which kiss the foul street as they are dragged over it. She has seen the wrong which she is doing to herself by pinioning her hands so that no motion can come to them as she walks. So she has cut four or five inches from the bottom of her walking-dress, and now goes forth, joyous in her exhiliration and proud of her freedom. In-doors she wears her long skirts and suffers not; in her daily out-door walks, she secures all the good to all the tissues of the body which unincumbered walking can provide.

If Weston were compelled to carry one hand behind him grasping a heavy skirt, he could not make more than one hundred miles a week. If he did not use his arms freely, in his walks, he would speedily become exhausted. Is it to be supposed that a woman can use her best efforts when hampered and clogged? Can she be strong while all her movements are constrained, and tend to weakness and inertia? Shall we not then insist upon daily exercise for women, under the most favorable conditions, free from restraint, absolved from the costume which encumbers and enthralls?

